# Trophic structure and origin of resources of soil macrofauna in the salt marsh of the Wadden Sea: a stable isotope (^15^N, ^13^C) study

**DOI:** 10.1186/s12862-022-02039-0

**Published:** 2022-06-27

**Authors:** Maria Rinke, Philipp M. Bendisch, Mark Maraun, Stefan Scheu

**Affiliations:** 1 J.F. Blumenbach Institute of Zoology and Anthropology, Animal Ecology, University of Göttingen, Untere Karspüle 2, 37073 Göttingen, Germany; 2 University of Göttingen, Centre of Biodiversity and Sustainable Land Use, Büsgenweg 1, 37077 Göttingen, Germany

**Keywords:** Salt marsh, Spatiotemporal dynamics, Trophic structure, Allochthonous resources, Autochthonous resources, Soil food web

## Abstract

Salt marshes exist along the gradient of the marine mudflat to the terrestrial dunes, with a gradient of shore height and associated plant zonation. The lower salt marsh (LSM) extends from the mean high tidal level to 35 cm above that level and is followed by the upper salt marsh (USM). Despite changes in the amount of allochthonous marine input and in abiotic conditions, little is known about changes in the trophic structure and used of basal resources by the soil macrofauna along marine—terrestrial boundaries. Natural variations in carbon stable isotope ratios (δ^13^C signatures) allow insight into basal resources of consumers such as marine algae, terrestrial C3 and C4 photosynthesising plants. Furthermore, variations in nitrogen stable isotope ratios (δ^15^N signatures) allow insight into the trophic position of consumers. We investigated spatial and temporal changes in stable isotope signatures in salt marsh soil macrofauna of the island of Spiekeroog, German Wadden Sea. The range of δ^15^N signatures indicated no changes in food chain length across salt marsh zones with consumers in both zones comprising primary decomposers, secondary decomposers and first order predators. However, the trophic position of individual species changed between zones, but in particular with season. Contrasting δ^15^N signatures, the range in δ^13^C signatures in the LSM was twice that in the USM indicating a wider range of resources consumed. Bayesian mixing models indicated predominant autochthonous resource use in both the LSM and USM, with the use of marine allochthonous resources never exceeding 29.6%. However, the models also indicate an increase in the use of marine resources in certain species in the LSM with no use in the USM. Overall, the results indicate that the resource use of salt marsh macrofauna varies more in space than in time, with the food web being generally based on autochthonous rather than allochthonous resources. However, there also is trophic plasticity in certain species across both temporal and spatial scales including variations in the use of allochthonous resources. Generally, however, marine input contributes little to the nutrition of salt marsh soil macroinvertebrates.

**Supplementary Information:**

The online version contains supplementary material available at 10.1186/s12862-022-02039-0.

## Introduction

Ecosystems are defined by primary production and the use of those autochthonous resources by consumers such as phytophagous animals or decomposers [1, 2]. Such systems include e.g., forests and eutrophic lakes [2, 3]. By contrast, habitats such as glacier forelands, beaches and most freshwater systems are characterized by the input of external, allochthonous resources [4–6]. This input of allochthonous resources may come from far away by wind, e.g. in glacier forelands, or from adjacent habitats with greater primary productivity, e.g. marine input on beaches [4, 5, 7]. Additionally, some systems receive both allochthonous and autochthonous resources, such as salt marshes.

Salt marshes are located at the interface between marine and terrestrial systems. As a result, they are regularly flooded resulting in a vegetation zonation associated with soil accumulation [8–10]. Salt marshes fulfil several important ecosystem services, such as shoreline protection from wave action and absorption of floodwaters during storms [11]. In addition, they are among the most productive systems of the world and act as carbon sink due to anoxic conditions in the soil [11]. Furthermore, they serve as refuge for juvenile fish and migratory birds [11]. Wadden Sea salt marshes are inhabited by a variety of vascular plants, such as *Elymus athericus* (*Elytrigia atherica*), *Atriplex portulacoides, Puccinellia maritima, Salicornia stricta* and *Spartina anglica*, contributing to high primary productivity [12, 13]. Additionally, tidal movements regularly flood parts of the marsh, depositing living and dead marine algae in the salt marsh [10]. In the North Sea, the tide transports large amounts of microalgae to the seashore including diatoms and dinoflagellates [14]. Both macroalgae and microalgae may flush into the marsh [9, 15], where they serve as resources for marine grazers occupying the lowest reaches of the marshes [16–19]. Despite this influx of marine resources to salt marshes, the relative importance of these allochthonous resources for consumers has hardly been studied.

The trophic structure of a food web determines the rate of energy fixation and transfer to higher levels in an ecosystem [20]. The trophic structure of food webs indicates its resilience to disturbances through coexisting species maintaining their trophic function and energy channelling [21–24]. Energy channels centre around basal resources such as primary producers or detritus [1, 23]. Additionally, resources from adjacent systems may affect higher trophic levels through improved primary production and associated secondary production [1, 4, 23]. These energy channels may vary in time and space, similar to the use of marine resources by terrestrial detritivores in salt marsh systems, which may vary across successional stages [13, 23]. Changes in energy channels, either through loss of a resource or addition of a new resource, influence the functional groups associated with those channels [1]. Therefore, a thorough understanding of the basal resources and the pathways they are channelled through food webs is vital to understand the functioning of the system.

Soil macrofauna includes species of a body size > 2 mm ranging from beetles to earthworms [25]. In the soil, macrofauna species are often considered ecosystem engineers because of their influence on the microbial, chemical and physical composition of the soil matrix [25–27]. Furthermore, by breaking down litter, they increase the rate of decomposition and nutrient cycling [25, 28]. The terrestrial macrofauna living in salt marshes may benefit from both autochthonous vascular plants and allochthonous algal or marine litter. This benefit may induce greater consumer production, thus influencing trophic structure [4]. Past studies of salt marsh succession by [8] as well as [23] suggest a decline in the use of marine resources with declining inundation frequency as a result of shore height. Trophic interactions, such as consumption of larval instars of rove beetles by the carabid beetle *Dicheirotrichus gustavii* [29] as well as consumption of algal wrack by the talitrid amphipod *Talitrus saltator* on the beaches of barrier islands [4, 30], have been identified. While studying trophic interactions by direct observations is difficult due to the small size of the soil fauna and their prey, and the inaccessibility of their habitat, past studies of forest soils have suggested that food specialists are rare [26, 31]. Given the increased availability of marine resources in the lower reaches of the salt marsh and the generalist feeding nature of soil decomposers, it is likely that these allochthonous resources are exploited influencing the trophic structure and nutrient availability in the marsh. However, the trophic structure of the soil macrofauna, their resource-use and changes across Wadden Sea salt marsh zones and seasons have not been investigated.

Given the regular flooding of the marshes and the small size of consumers and prey, delineating the trophic structure of salt marsh food webs using direct observations of trophic interactions is virtually impossible. However, for studying the trophic structure of animal communities natural variations in stable isotope ratios (^15^N/^14^N, ^13^C/^12^C) are increasingly used and this also applies to salt marshes [10]. Previous studies in the salt marsh of the North Sea using stable isotopes in fact indicated increased use of marine resources by oribatid mites at the lowest reaches of the salt marsh [9]. In addition, studies based on stable isotopes allowed insight into the diet and trophic position of benthic macrofauna of salt marshes [32, 33]. Stable isotope analysis allows determining trophic levels through ^15^N/^14^N ratios as well as identification of the resources used through ^13^C/^12^C ratios [34]. ^15^N concentrations increase with transfer to higher trophic levels, thus allowing to estimate the trophic level of consumers [34, 35]. ^13^C concentration, on the other hand, varies between resources, specifically plants with different photosynthetic pathways (C3 and C4) [34, 35] allowing to trace the channelling of these resources through food webs. Both of these isotopes have been used in intertidal salt marshes to understand changes in the quality of organic matter with time [36]. In addition, they have been used to delineate marine resource use by insect larvae consumed by spiders on shorelines of the Baltic Sea [37] as well as by Polychaeta, Pulmonata and Amphipoda of coastal waters [33, 38]. δ^13^C signatures become especially useful when investigating the use of marine and terrestrial resources due to the distinct δ^13^C signatures of algae [9, 23, 33]. Differences in δ^13^C signatures between algae and other plants are due to algae using bicarbonate as inorganic carbon source resulting in enriched δ¹³C signatures, which may also vary with light and nutrient availability [39–41]. Therefore, using δ^13^C and δ^15^N signatures may allow to discern the use of allochthonous marine and autochthonous terrestrial plant resources as well as the trophic structure of salt marsh soil macrofauna.

Here, we investigated the importance of allochthonous marine input (mainly algae) compared to autochthonous terrestrial resources for the soil macrofauna of the upper (USM) and lower salt marsh (LSM). Furthermore, we compared the trophic structure of the salt marsh soil macrofauna across salt marsh zones as well as seasons. More specifically, we investigated the following hypotheses: (1) The consumption of allochthonous marine algal resources increases with greater tidal influence i.e., the enrichment of ¹³C in consumers of the LSM is higher than in consumers of the USM. (2) Resembling terrestrial habitats, salt marsh soil macrofauna communities consist of four trophic levels including primary decomposers, secondary decomposers, first order predators and second order predators [26]. (3) Resource use of soil macrofauna varies among seasons (spring, summer, autumn) and zones with allochthonous marine resources being especially important for the salt marsh macrofauna food web in autumn due to heavy storms carrying large amounts of marine resources to higher positions in the salt marsh.

## Materials and methods

### Study site

Sampling was performed in the Wadden Sea salt marsh of Spiekeroog, Lower Saxony, Germany, as part of the DynaCom project (https://uol.de/en/icbm/collaborative-projects/dynacom). The Wadden Sea stretches across The Netherlands to Denmark and holds a vast area of salt marshes and mudflats [42, 43]. Back-barrier marshes form on the leeward side of barrier islands, thus are sheltered from strong wave action [8]. Due to the lack of strong wave action, sediment is deposited and over time a gradient of shore height is formed [8, 10]. This gradient alters the inundation period and frequency, resulting in different vegetation zones. The USM is located 35 cm or more above the mean high-water level, with inundations occurring up to 70 times a year, whereas the LSM lies between 0 and 35 cm above the mean high-water level and is flooded up to 250 times a year [43, 44].

### Sampling

Sampling was performed on the 16th of April (spring), 16th of July (summer) and 22nd of October 2019 (autumn) during low tide along five transects (53°45′2″−53°47′1″N, 7°40′0″−7°49′1″E). Per transect and zone one soil core (ø 20 cm, depth 10 cm) was taken. Animals were extracted by heat [44] and stored in 70% ethanol at − 20 °C. Animals were determined to group or species level [45]. Additionally, litter, soil, vascular plant species and macroalgae were collected by hand and stored at − 20 °C (Additional file 1:  Table S2).

### Sample preparation and stable isotope analysis

Taxa/species were not present in all soil cores, we attempted to analyse at least three specimens per taxon/species and sampling date originating from separate soil cores. Samples were dried at 60 °C for 24 h and weighed into tin capsules using a fine scale (Cubis MSE 3.6P, Sartorius). Variations in stable isotope ratios (^13^C/^12^C and ^15^N/^14^N) were measured by an elemental analyser (Euro EA 3000, EuroVector S.p.A; Milano Italy) modified for small samples coupled with an isotope mass spectrometer (Delta V Plus, Thermo Electron, Bremen Germany) [46]. Ratios of stable isotopes were expressed as δX (‰) = [(R_sample_ − R_standard_)/R_standard_] × 1000, with ‘X’ representing the target isotope and ‘R’ the heavy-to-light isotope ratios (^13^C/^12^C and ^15^N/^14^N) of the sample and standard, respectively. Vienna PD Belemnite (PDB) was used as standard for δ^13^C and atmospheric nitrogen for δ^15^N. Acetanilide was used for internal calibration.

### Statistical analysis

Signatures of δ^15^N and δ^13^C of macrofauna taxa were analysed using linear mixed effects models in R (4.1.0) (R. core Team 2021) using the packages emmeans (Version 1.6.2-1), lme4 (Version 1.1-27.1), car (Version 3.0-11), lmerTest (Version 3.1-3) and dplyr (Version 1.0.7). “Zone” and “Season” were included as fixed factors and “Core ID” nested within “Transect” as random factor. Due to inconsistent occurrence across zone and season, macrofauna taxa were analysed separately. The full factorial model with Zone and Season could only be fitted for *T. saltator* (Amphipoda), *Ochthebius dilatatus* (Coleoptera, Hydraenidae) and *D. gustavii* (Coleoptera, Carabidae) present in both zones and all three seasons, except for *D. gustavii* which was not found in the USM in April. Staphylinidae larvae (April, July and October) and chalcidoid wasps (Chalcidoidea) (July and October) were only found in the LSM, and therefore only season was fitted as fixed factor. Similarly, in *Amischa* spp. (Coleoptera, Staphylinidae) only season was fitted as fixed factor as it was only found in the USM (April, July and October). To inspect variations in the length of the food chain as well as the span of resources with Season and Zone we calculated ^15^N and ^13^C ranges in cores where two or more species occurred as the difference between the most ^13^C or ^15^N enriched and the least ^13^C or ^15^N enriched taxa/species, and analysed the data using MANOVA in Statistica 13 (TIBCO Software Inc. 2018; http://tibco.com). To determine the dependence of species on autochthonous and allochthonous resources, Bayesian mixing models were calculated per season using FRUITS (Beta Version 2.1.1) using fractionation factors for ^15^N and ^13^C of 3.4‰ and 4.0‰, respectively. The fractionation factors are well established; for ^13^C the fractionation factor is based on the ‘detrital shift’ as described in [47] and reflects the relative enrichment of soil animals compared to litter. For ^15^N it reflects the average trophic level fractionation [47, 48]. For the first trophic level mean δ^15^N of litter ± 1.7‰ was used. Because of lack of replicates across season for *Dyschirius* sp. (Coleoptera, Carabidae), *Argenna* sp. (Araneae, Dictynidae) and Linyphiidae (Araneae), mixing models analysing variations in stable isotope signatures in these taxa/species were only fitted with Zone as fixed factor. The vegetation at both zones was dominated by C3 plants and the mean signatures of algae and vascular plant species were used in the mixing models (Additional file 1: Table S1). The C4 plant *Spartina anglica*, present at the transition to the mudflats, was not included in the pool of potential resources as animal signatures indicated that it did not form part of the basal resources.

## Results

**Fig. 1:**
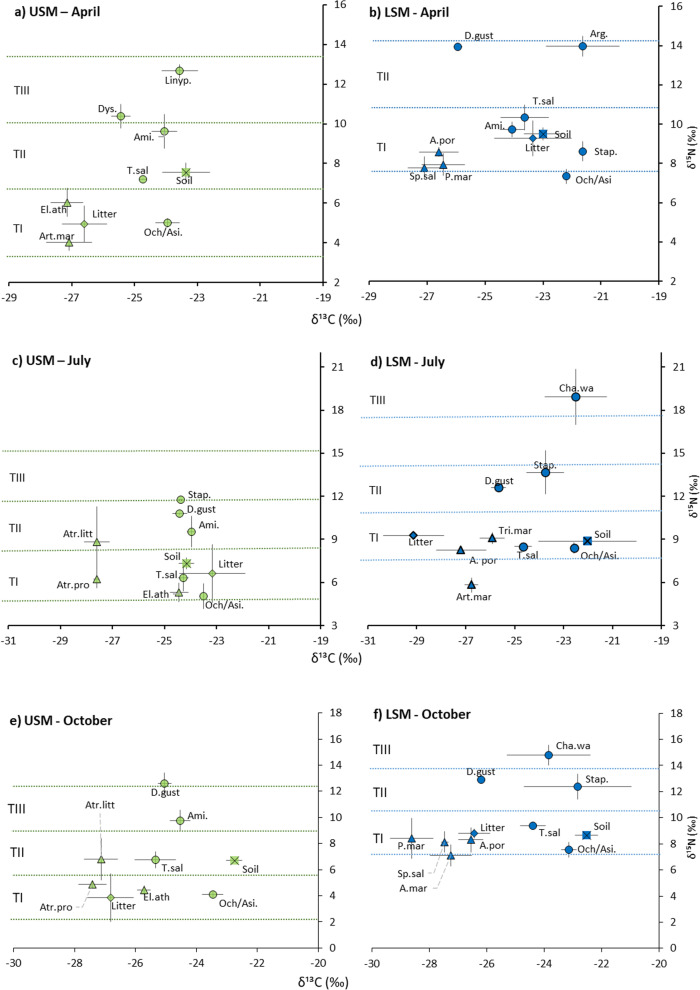
Scatterplot of δ^15^N und δ^13^C signatures of salt marsh fauna taxa / species (circles), plants (triangles), litter (diamonds) and soil (crossed squares) in the upper salt marsh (USM)= green and lower salt marsh (LSM)= blue; (**a**) USM in April, (**b**) LSM in April, (**c**) USM in July, (**d**) LSM in July, (**e**) USM in October and (**f**) LSM in October; means ± SD. Dotted lines indicate trophic levels. Animals: Ami.—*Amischa* sp., Arg.—*Argenna* sp., D. gust—*Dicheirotrichus gustavii*, Dys.—*Dyschirius* sp., Ochth.—*Ochthebius dilatatus*, Linyp.—Linyphiidae, Stap.—Staphylinidae larvae, T. sal—*Talitrus saltator*. Plants: Atr.litt—*Atriplex littoralis*, Art.mar—*Artemisia maritima*, A. por—*Atriplex portulacoides*, Atr.pro—*Atriplex prostrata*, El.ath—*Elymus athericus*, P.mar—*Puccinellia maritima*, Sp.sal—*Spergularia salina*

In total, six macrofauna species, 20 plant species (Additional  file 1: Table S2) as well as soil and litter were collected and analysed for δ^13^C and δ^15^N signatures. In April, animal δ^13^C signatures ranged from − 21.63 to − 25.93‰ and δ^15^N signatures from 5.02 to 13.97‰, respective ranges in July were − 22.50 to − 25.65 for δ^13^C and 5.05 to 18.93‰ for δ^15^N, and in October − 22.83 to − 26.19‰ for δ^13^C and 4.10 to 14.79‰ for δ^15^N (Fig. 1). Respective ranges of terrestrial vascular plants for δ^13^C and δ^15^N were − 27.15 to − 26.44‰ and 4.01 to 10.44‰ in April, − 27.61 to − 24.44‰ and 5.32 to 9.08‰ in July, and − 28.62 to − 25.72‰ and 4.43 to 8.42‰ in October (Fig. 1). Typically, the communities spanned three trophic levels, including primary decomposers, secondary decomposers and first order predators (Fig. 1). Generally, the range in δ^15^N signatures neither varied significantly between zones nor among seasons and averaged 5.71 ± 2.43‰. By contrast, the range in δ^13^C signatures varied significantly with Zone, with 0.91 ± 0.40‰ in the USM and 2.67 ± 0.43‰ in the LSM (F_1,18_= 15.26, p = 0.001), but not with Season.

### Salt marsh zones

Among individual taxa δ^15^N signatures between the LSM and USM were significant in *O. dilatatus* (7.81 ± 0.69‰ and 4.68 ± 0.70‰, respectively) and *T. saltator* (9.54 ± 0.95‰ and 6.70 ± 0.82, respectively) (Table 1). Further, δ^13^C signatures of *O. dilatatus* were significantly higher in the LSM than the USM (− 22.69 ± 0.45‰ and − 23.65 ± 0.41‰, respectively), whereas in *D. gustavii* δ^13^C signatures in the LSM were significantly lower than in the USM (− 25.92 ± 0.35‰ and − 24.73 ± 0.40‰, respectively).

**Table 1:** F- and p-values of linear mixed-effects models on variations in δ^15^N and δ^13^C signatures of macrofauna taxa/species with season (April, July, October) and salt marsh zone (upper salt marsh, lower salt marsh) on the island of Spiekeroog in 2019

Species	n	Zone (Z)				Season (S)				Z × S			
		ẟ^15^N		ẟ^13^C		ẟ^15^N		ẟ^13^C		ẟ^15^N		ẟ^13^C	
		F	p	F	p	F	p	F	p	F	p	F	p
*Talitrus saltator*	15	**26.19**	**0.004**	2.40	0.156	**10.76**	**0.019**	1.23	0.336	0.53	0.620	1.71	0.235
*Ochthebius dilatatus*	16	**61.01**	**0.001**	**7.73**	**0.003**	**7.47**	**0.039**	1.55	0.269	1.05	0.426	**7.84**	**0.013**
*Dicheirotrichus gustavii*	10	9.22	0.075	**37.44**	**0.001**	**9.85**	**0.021**	**8.97**	**0.024**	4.95	0.069	0.06	0.819
Chalcid wasps	6	Only LSM				11.19	0.075	0.95	0.384	N/A			
Staphylinidae larvae	7					**10.99**	**0.015**	2.64	0.275				
*Amischa* spp.	9	Only USM				0.41	0.705	**27.33**	**0.030**				

‘n’ number of replicates, *N/A* not analysed

Bold values indicate *p* = < 0.05

### Season

δ^15^N signatures among individual species, only varied significantly with season in *D. gustavii*, *O. dilatatus*, Staphylinidae larvae and *T. saltator* (Table 1). Seasonal changes are presented separately for individual taxa/species because no common patterns were evident. In *D. gustavii* δ^15^N signatures were significantly higher in October (12.89 ± 0.64‰) than in July (11.89 ± 1.02‰), whereas in *O. dilatatus* they increased significantly from April (5.95 ± 1.32‰) to July (7.06 ± 1.96‰) and decreased again in October (5.82 ± 1.94‰). In Staphylinidae larvae δ^15^N signatures significantly increased from April (8.62 ± 0.62‰) to July (13.66 ± 2.15‰) and remained at a similar level in October (12.36 ± 1.18‰). In *T. saltator* δ^15^N signatures declined from April (9.09 ± 1.61‰) to July (7.20 ± 1.28‰) and remained at a similar level in October (7.79 ± 1.38‰). δ^13^C signatures varied significantly with season in *Amischa* sp. and *D. gustavii.* Furthermore, in *O. dilatatus* the effect of Season depended on Zone (Table 1). In *Amischa* sp. δ^13^C signatures increased from April (− 24.05 ± 1.04‰) to July (− 23.97 ± 1.32‰) and declined again in October (− 24.52 ± 1.01‰). Also, in *D. gustavii* δ^13^C signatures decreased from July (− 25.16 ± 0.65‰) to October (− 25.73 ± 0.75‰). By contrast, in *O. dilatatus* ẟ^13^C signatures changed little with Season in the USM (− 23.95 ± 0.46, − 23.51 ± 0.01‰ and − 23.46 ± 0.42‰ for April, July and October, respectively), whereas in the LSM ẟ^13^C signatures gradually declined from April (− 22.19 ± 0.12‰) to July (− 22.57 ± 0.14‰) to October (− 23.14 ± 0.32‰).

### Allochthonous resource use

**Fig. 2:**
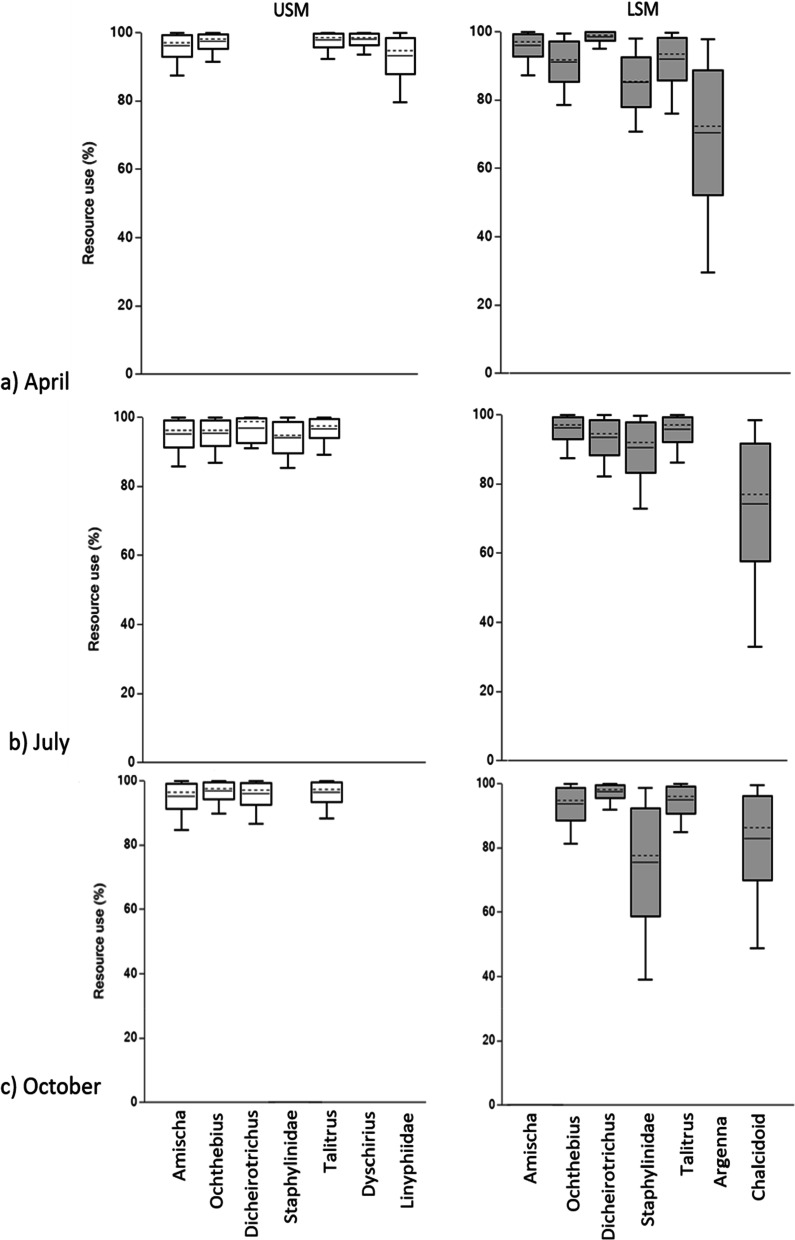
Boxplot of results of Bayesian mixing models on the use of terrestrial vs. marine resources (%) by macrofauna species in the salt marsh of Spiekeroog in (**a**) April, (**b**) July and (**c**) October. Left panel (white boxes) = upper salt marsh (USM), right panel (grey boxes) = lower salt marsh (LSM). Boxes represent the 68% confidence intervals, error bars 95% confidence intervals; the solid horizontal line represents the mean, the dashed horizontal line the median

Bayesian mixing models indicated an almost exclusive use of terrestrial resources across taxa in both the LSM and USM. In April they used 85–98% terrestrial resources with the exception of *Argenna* sp. in the LSM (70.4 ± 17.8%) (Fig. 2a); in July they used 90–97% terrestrial resources with the exception of the chalcidoid wasps of the LSM (74.4 ± 17.3%) (Fig. 2b). In October they used 83–97% terrestrial resources with the exception of Staphylinidae larvae in the LSM (75.6 ± 16.2%) (Fig. 2c).

## Discussion

We investigated the trophic structure and allochthonous resource use of the soil macrofauna of the Wadden Sea salt marsh. Consisting of only three trophic levels the macrofauna food web is simpler than the previously investigated mesofauna food web [10]. Further, the range of δ^13^C signatures in the LSM significantly exceeded that in the USM indicating a wider range of basal resources used in the LSM than in the USM. However, as indicated by Bayesian mixing models, allochthonous marine resources generally are of little importance for the nutrition of soil macrofauna in both the LSM and USM. Thereby, despite the proximity of marine and terrestrial habitats, we found little evidence for the use of marine resources by the soil macrofauna.

### Differences between salt marsh zones

Stable isotope signatures of soil macrofauna taxa/species differed significantly between the USM and LSM. δ^15^N signatures were generally higher in the LSM compared to the USM and this applied in particular to *O. dilatatus* (Coleoptera, Hydraenidae) and *T. saltator* (Amphipoda) as well as in trend to *D. gustavii* (Coleoptera, Carabidae). Only δ^13^C signatures varied significantly between salt marsh zones in *D. gustavii*, with higher enrichment in the USM than the LSM, contradicting our first hypothesis. Higher δ^15^N signatures in macrofauna taxa/species in the LSM than in the USM contrast patterns in the salt marsh mesofauna, in which δ^15^N signatures were higher in the USM than in the LSM [10]. Considering the higher δ^15^N signatures of soil, plant and litter material in the LSM than the USM, the higher signatures of *O. dilatatus* and *T. saltator* in the LSM than in the USM are likely related to their basal resources, pointing to the predominant use of autochthonous resources. In *D. gustavii* δ^15^N signatures did not differ significantly between the USM and LSM. However, in the USM *D. gustavii* occupied a higher trophic position than in the LSM, as discussed in [47], high δ^15^N and low δ^13^C signatures indicates consumption of resources based on freshly fixed carbon or consumers thereof, again pointing to the dominant use of autochthonous resources.

### Trophic level

Regardless of season or zone, macrofauna communities consisted of three trophic levels rejecting our second hypothesis. The results suggest that the salt marsh macrofauna food web uniformly comprises primary decomposers, secondary decomposers and first order predators. The latter included chalcidoid wasps (Chalcidoidea) parasitizing a wide range of host species [49]. These findings are in contrast to [10] reporting four trophic levels for the salt marsh soil mesofauna. The lower number of trophic levels in macrofauna across both zones is likely due to high disturbance by inundation reducing soil macrofauna diversity. As proposed by the intermediate disturbance hypothesis, we expected a reduction in diversity with disturbance frequency [50]. However, while inundation frequency indeed is lower in the USM than in the LSM, soil salinity is high in both the USM and LSM [51, 52]. As discussed by [50] increased abiotic stressors may reduce diversity and favour the dominance of specialists. Contrasting these assumptions, the wider range in δ¹³C signatures of macrofauna species in the LSM compared to the USM points to more flexible resource use in species of the former. Overall, the results suggest that the salt marsh soil macrofauna food web is simpler than expected, presumably due to frequent flooding and associated disturbances.

### Season

Although seasonal variations in stable isotope signatures generally were less pronounced than variations between salt marsh zones, δ^15^N signatures varied significantly with season in *D. gustavii*, *O. dilatatus*, Staphylinidae larvae and *T. saltator*. Further, δ^13^C signatures varied significantly in *D. gustavii*, *Amischa* sp. (Coleoptera, Staphylinidae) and *O. dilatatus*, but in the latter this depended on salt marsh zone. In *D. gustavii* and *Amischa* sp. δ^13^C signatures declined from July to October, whereas in *O. dilatatus* they declined from April to October but only in the LSM, contradicting our third hypothesis. δ^15^N signatures in *D. gustavii* of the USM changed considerably with season indicating a switch from living as secondary decomposer in April to living as predator in October, whereas in the LSM *D. gustavii* constantly lived as secondary decomposer. Changes in resource use in *D. gustavii* in the USM is supported by the decline in δ¹³C signatures from July to October indicating a switch from decomposer prey in July to herbivore prey in October [47]. In *O. dilatatus*, δ^15^N signatures in the LSM, but not the USM, were higher in July than in April and October. Further, δ^13^C signatures in the LSM declined later in the year, indicating increased use of autochthonous terrestrial resources. Previous studies showed *Ochthebius* species to scrape mats of microalgae on rocky shores as well as to feed on detritus [53, 54]. In the salt marsh *Ochthebius* species occur in or near saline channels, with larval stages living submerged [55–57] suggesting that they consume resources of marine origin. Contrasting this assumption, our results based on Bayesian mixing models indicate that the resources used by *O. dilatatus* in salt marshes are primarily based on autochthonous vascular plant litter material. δ^15^N of Staphylinidae larvae in the LSM point to a trophic change from living as primary decomposer in April, to living as secondary decomposer in July and October. These changes could point to increased consumption of partially decomposed litter colonized by microorganisms, or to later larval stages living as predators by feeding on primary decomposers. Variations in δ^15^N signatures with season in *T. saltator* indicate trophic plasticity in this species in particular in the USM, where its trophic position changed from secondary decomposer in April to primary decomposer in July and back to secondary decomposer in October. Direct feeding on plant litter might be related to increased plant growth and litter production during summer. Overall, variations in δ^15^N signatures in *T. saltator* suggests that in salt marshes this species lives as opportunistic omnivore with its diet including microorganisms but also plant litter material depending on resource availability. This contrasts previous studies at beaches suggesting that *T. saltator* predominantly feeds on algae [7]. In *Amischa* sp. from the USM, δ^13^C signatures declined significantly in October, which may point to increased predation on herbivores, whereas in April and July they may feed more on decomposer prey [47]. Overall, resource use of a number of salt marsh macrofauna species changed with season indicating trophic plasticity. However, this was restricted to few species suggesting that overall, the resources used vary little between seasons.

### Allochthonous resource use

As indicated by Bayesian mixing models the salt marsh macrofauna almost exclusively exploits autochthonous C3 plant resources with the use of allochthonous marine resources being restricted to few species and not exceeding 29.6%, contradicting our third hypothesis. Conversely suggesting that despite salt marshes being sinks for marine carbon [58] carbon of marine origin is not a predominant resource for the salt marsh soil macrofauna. Species feeding predominantly on terrestrial resources regardless of season included *D. gustavii*, *Amischa* sp. and *T. saltator*. In the carabid beetle *D. gustavii* this suggests predation on phytophagous species. By contrast, *Amischa* sp. likely fed on both animal prey as well as fungi as Aleocharinae rove beetles are known to function as both detritivores and predators [59]. As indicated by their δ^15^N signatures, in the salt marsh they predominantly function as detritivores of terrestrial C3 plant-based resources, only occasionally feeding on animal prey. Unexpectedly, *T. saltator* also exclusively relied on terrestrial resources (see above), with its variable trophic position indicating that in salt marshes it lives as omnivore. On the other hand, the spider *Argenna* sp. (Araneae, Dictynidae), Staphylinidae larvae and chalcidoid wasps indicated some marine resource use at least at certain sampling dates. In April, the use of marine resources was highest in *Argenna* sp. in the LSM, presumably due to feeding on dipterans with marine larval stages [37]. In Staphylinidae larvae the use of marine resources in the LSM was highest in October; in chalcidoid wasps in April and July. These resource changes may be related to higher flooding frequency at those time periods and the associated increase in marine detritus. Staphylinidae larvae may either use fungi in the deposited detritus or prey on detritivores such as dipteran larvae. Similarly, increased use of marine resources in chalcidoid wasps, might be due to parasitizing dipteran larvae developing in deposited marine detritus as has been shown for seaweed flies on beaches [60]. Overall, the results suggest that allochthonous input of marine resources is of limited importance for the salt marsh macrofauna community regardless of season and zone.

## Conclusions

We investigated the trophic structure, trophic plasticity and resource use of the salt marsh macrofauna along spatial and temporal scales. Compared to the soil mesofauna and typical terrestrial habitats, the trophic structure of the salt marsh macrofauna is more simple with only three trophic levels irrespective of zone and season, presumably reflecting disturbance frequency and harsh abiotic conditions resulting in less diverse and trophically less structured communities. δ^13^C signatures and Bayesian mixing models indicated that the macrofauna community predominantly relies on autochthonous C3 plant-based resources, regardless of season and zone. Changes in resource use were species-specific with the use of allochthonous marine-based resources being restricted to chalcidoid wasps, the spider *Argenna* sp. and Staphylinidae larvae. Generally, variations in stable isotope ratios suggest that spatial variation in trophic niches exceeds seasonal variation. Overall, the trophic structure of the salt marsh macrofauna is rather simple, similar to arable systems, with terrestrial C3 plants as the main resource and allochthonous marine material being of little importance. Therefore, despite their importance as blue carbon systems, the soil macrofauna of salt marshes has little association with marine carbon.

## Supplementary information

**Additional file 1.** Summary table of resource isotope values used for each Bayesian mixing model. Separated into Marine - meaning algae - and terrestrial - meaning vascular C3 plant species. Terrestrial vascular plants were collected across the USM - upper salt marsh and LSM - lower salt marsh. For the Bayesian mixing model only overall mean values of marine resources and terrestrial resources were used.

## Data Availability

The datasets generated and/or analysed during the current study are available in the DRYAD repository (doi:10.5061/dryad.tdz08kq1t), https://datadryad.org/stash/share/sCK5KbHOxJzZgmCQsrYkdtJiZhH10igspyu2800HBzk.
